# Human male infertility and its genetic causes

**DOI:** 10.1002/rmb2.12017

**Published:** 2017-03-26

**Authors:** Toshinobu Miyamoto, Gaku Minase, Takeshi Shin, Hiroto Ueda, Hiroshi Okada, Kazuo Sengoku

**Affiliations:** ^1^ Department of Obstetrics and Gynecology Asahikawa Medical University Asahikawa Japan; ^2^ Department of Urology Dokkyo Medical University Koshigaya Hospital Koshigaya City Japan

**Keywords:** azoospermia, male infertility, mutation, *PLK4*, *SYCP3*

## Abstract

**Background:**

Infertility affects about 15% of couples who wish to have children and half of these cases are associated with male factors. Genetic causes of azoospermia include chromosomal abnormalities, Y chromosome microdeletions, and specific mutations/deletions of several Y chromosome genes. Many researchers have analyzed genes in the AZF region on the Y chromosome; however, in 2003 the *SYCP3* gene on chromosome 12 (12q23) was identified as causing azoospermia by meiotic arrest through a point mutation.

**Methods:**

We mainly describe the *SYCP3* and *PLK4* genes that we have studied in our laboratory, and add comments on other genes associated with human male infertility.

**Results:**

Up to now, The 17 genes causing male infertility by their mutation have been reported in human.

**Conclusions:**

Infertility caused by nonobstructive azoospermia (NOA) is very important in the field of assisted reproductive technology. Even with the aid of chromosomal analysis, ultrasonography of the testis, and detailed endocrinology, only MD‐TESE can confirm the presence of immature spermatozoa in the testes. We strongly hope that these studies help clinics avoid ineffective MD‐TESE procedures.

## 1 Introduction

Infertility affects ~15% of couples who wish to have children and half of these cases are associated with male‐related factors.^1^ Genetic causes of azoospermia include chromosomal abnormalities, Y chromosome microdeletions, and specific mutations or deletions of several Y chromosomal genes.^2^, ^3^ Thus, in 1995, it was demonstrated that *DAZ* mutations (Yq11.23) cause various forms of human male infertility that range from oligospermia to azoospermia.^4^, ^5^ In 1997, it was reported that *RBMY* (Yq11.223) mutations cause azoospermia via meiotic arrest^5^, ^6^ and, in 1999, it was shown that *USP9Y* (Yq11.2) mutations lead to azoospermia that is secondary to hypospermatogenesis.^5^, ^7^ The azoospermia factor (AZF) region on the Y chromosome is one of the most intensively studied regions in male infertility.^8^, ^9^ However, no Y chromosome gene that causes male infertility directly has been found since 1999. In 2003, the authors found that a mutation of the human *SYCP3* gene at 12q23 causes azoospermia by meiotic arrest.^10^ Since then, many researchers worldwide have analyzed mutations in other autosomal genes that might cause male infertility. In this review, the *SYCP3* and *PLK4* genes that have been studied in the authors’ laboratory are mainly described and comments on other genes that are associated with human male infertility have been added.

## 2 Culprit Genes That Have Been Identified in Autosomes

The three genes that were mentioned above are typical ones that are involved in spermatogenesis and are localized to the AZF region on the Y chromosome. Although many clinicians and researchers have analyzed this region and striking advances have been made in molecular genetics, the last such gene to be identified was *USP9Y* in 1999. Analyses using gene knockout mice have shown that there are numerous autosomal genes controlling spermatogenesis and that meiosis—indispensable for spermatogenesis—is also essential for oogenesis. Consequently, the authors proceeded with an analysis based on the hypothesis that genes with mutations leading to human azoospermia are also present on autosomes.

### 2.1 *SYCP3* and azoospermia by meiotic arrest

In 2000, a *Sycp3* (*Scp3*) gene knockout mouse was reported.^11^ SYCP3 (synaptonemal complex protein 3) is a DNA‐binding protein that is related to the synapses involved in germ cell meiosis.^12^, ^13^, ^14^ Both the male and female *Sycp3* knockout mice are developmentally normal. However, the homo‐mutant male has no reproductive potential. An analysis of these knockout mice showed the testes to be markedly smaller than normal and histology showed meiotic arrest, with a complete absence of round and elongated spermatids, which typically appear after meiosis. The male mice had no mature spermatozoa.^11^ When compared with wild‐type mice, the female *Sycp3* gene knockout mice produced fewer offspring, even though they were capable of gestation and parturition.^11^ A subsequent detailed analysis showed the cause to be fetal death in utero that is caused by a chromosomal aberration. The frequency of fetal death in utero increased with the age of the mouse.^15^ Based on the hypothesis that the human *SYCP3* gene might play an important role in human spermatogenesis, as in mice, the authors began an analysis of human genes.

The authors isolated human *SYCP3* cDNA and found that it is located on chromosome 12 (12q23). Its expression is specific to the testis.^16^ It encodes 236 amino acids and has two coiled‐coil domains.^10^ A mutation analysis was performed for all of the coding regions and adjacent introns in 19 patients with azoospermia that had been diagnosed histologically as being caused by a meiotic anomaly. The analysis detected a heterozygous deletion of one adenosine base at a 643‐nucleotide site in two of the 19 patients. In order to rule out gene polymorphisms, a sequence analysis also was performed by using DNA from 75 healthy men. No mutation was detected in any of these men (*P*=.039).^10^ It has been known for some time that a coiled‐coil domain that is present in three regions of the rat *Sycp3* gene plays an important role in protein binding.^16^ The deletion that was detected in the authors’ investigation was present in the coiled‐coil domain. The mutation results in a frame shift and an early stop codon appears, resulting in an incomplete domain. Consequently, a functional analysis of the mutation was carried out. The truncated human *SYCP3* with a 643 delA mutation has little functional protein–protein interaction.^10^

Based on these findings, the authors’ laboratory succeeded for the first time in identifying the azoospermia culprit gene, *SYCP3*, outside of the AZF region of the Y chromosome, on human chromosome 12.^10^ In 2009, it was reported that mutations in human *SYCP3* are associated with recurrent pregnancy loss.^17^ Two out of 26 women with recurrent pregnancy losses of unknown cause were found to carry independent heterozygous nucleotide alterations in *SYCP3*, neither of which was present among a group of 150 fertile women. An analysis of the transcripts from minigenes harboring each of these two mutations revealed that both affected normal transcriptional splicing, possibly resulting in the production of C‐terminally mutated proteins. The mutant proteins were found to interact with their wild‐type counterpart in vitro and to inhibit the normal fiber formation of the SYCP3 protein when coexpressed in a heterologous system. It is possible that the mutations generate an aberrant synaptonemal complex in a dominant‐negative manner and contribute to abnormal chromosomal behavior that might lead to recurrent miscarriage.^17^ These data suggest that mutations to *SYCP3* induce azoospermia by meiotic arrest in men and recurrent pregnancy losses in women.

### 2.2 KLHL10 and oligozoospermia

In 2006, a new approach was used to identify the gene mutations that are associated with male infertility.^18^ The feasibility of obtaining full‐length mRNAs from transcriptionally inert human spermatozoa in semen was explored as an uninvasive diagnostic tool for identifying germline mutations in candidate infertility‐associated genes. The efficacy of reverse‐transcription polymerase chain reaction (PCR) amplification on sperm RNA from infertile patients with wide‐ranging sperm concentrations varied between 91% and 99% for multiple haploid germ cell‐expressed genes. Using this method, seven oligozoospermic patients were identified with missense and splicing mutations in the germ cell‐specific gene, *KLHL10*, at 17q21.^18^ This is a human homolog of the *kelch* gene in *Drosophila melanogaster*.^19^ The mouse *Klhl10* is an essential gene for spermiogenesis and acts in a dosage‐sensitive manner. It was reported that heterozygosity for a null mutation in *Klhl10* is characterized by germ cell loss and the defective morphology of spermatids.^20^ Three of 270 severely oligozoospermic patients harbored *KLHL10* alterations, which were absent in 395 controls. Two *KLHL10* missense mutations (A313T and Q216P) resulted in impaired homodimerization with the wild‐type protein in yeast interaction assays, suggesting a functional deficiency. Thus, *KLHL10* mutations in oligozoospermic patients impair homodimerization.^18^ This was the second reported autosomal gene with mutations that were associated with human male infertility.

### 2.3 *SPATA16* and globozoospermia

Globozoospermia is rare (an incidence of <0.1% in male infertile patients) but there exists severe teratozoospermia, characterized by ejaculates consisting completely of round‐headed spermatozoa that lack an acrosome or, in partial globozoospermia, containing a variable proportion (20%‐90%) of acrosome‐less spermatozoa.^21^ Men who are affected with total globozoospermia are infertile and even the application of intracytoplasmic sperm injection (ICSI) has met with disappointingly low success rates.^21^ Globozoospermia originates from disturbed spermiogenesis and although the underlying cause is still unknown, a genetic contribution appears to be supported by several familial case reports^22^, ^23^, ^24^ and by three recessive mouse models.^25^, ^26^, ^27^ However, no causative gene mutation has been identified in these orthologs or in any other human gene. A family with three affected brothers was reported, in whom a homozygous mutation in the spermatogenesis‐specific gene, *SPATA16*, at 3q26, was identified.^28^ The first case of a successful pregnancy that was obtained by using ICSI for an infertile 29 year old man with a homozygous mutation in *SPATA16* has been reported.^29^

### 2.4 *AURKC* and large‐headed polyploid spermatozoa or macrozoospermia

A genome‐wide microsatellite scan was performed on 10 infertile men with a normal somatic karyotype but with spermatozoa that were characterized mainly by large heads, a variable number of flagella, and an increased chromosomal content.^30^, ^31^, ^32^, ^33^ A common region of homozygosity was identified that harbored the gene that encodes aurora kinase C (*AURKC*) at 19q13 in all of these men. It is known to be highly expressed in the testis^34^, ^35^ and is putatively involved in cytokinesis, mitosis, and meiosis.^36^, ^37^ A single nucleotide deletion (ca 144delC) was detected in the *AURKC* coding region in all of the patients who were analyzed.^33^ This founder mutation (L49Wfsx22) results in the premature termination of translation, yielding a truncated protein that lacks the kinase domain.^33^ Functional in vivo and ex vivo analyses were carried out and it was found that the truncated protein had lost its function. Thus, a homozygous mutation of *AURKC* yields large‐headed polyploid spermatozoa and causes male infertility. In addition, new *AURKC* mutations that cause macrozoospermia have been reported.^38^, ^39^

### 2.5 *HSF2* and idiopathic azoospermia

HSF2, belonging to the family of heat‐shock transcription factors (HSFs), plays a key role in regulating normal spermatogenesis in mice.^40^ At least two splice forms, HSF2a and HSF2b, were identified for HSF2 and the *Hsf2a* gene was expressed predominantly in the mouse testis.^41^ In response to various stimuli under normal physiological or stressed conditions, HSFs regulate the dynamic expression of different heat‐shock proteins (HSPs) that are responsible for subsequent downstream effects, including stress‐related cytoprotective functions, folding and assembling of nascent polypeptides, and intracellular transport of proteins.^42^ Similarly, a role has been suggested for HSF2 (6q22.31) in spermatogenesis by regulating the expression of different HSPs, as both *Hsf2*‐ and *Hspa2*‐knockout mice suffer from male reproductive defects.^43^, ^44^, ^45^, ^46^, ^47^, ^48^ Whether or not HSF2 is involved in the pathogenesis of human idiopathic azoospermia was investigated. All the exons of *HSF2* were sequenced in 766 patients who had been diagnosed with idiopathic azoospermia and 521 proven fertile men.^49^ A number of coding mutations that were limited to the patient group, including three synonymous mutations and five missense mutations, were identified.^49^ Of the missense mutations, a functional assay demonstrated that one heterozygous mutation, R502H, caused a complete loss of HSF2 function of the wild‐type allele through a dominant‐negative effect, thus leading to the dominant penetrance of the mutant allele.^49^ This study supports a role for HSF2 in the pathogenesis of idiopathic azoospermia in humans.

### 2.6 *SEPT12* and oligoasthenozoospermia or asthenoteratozoospermia

Septin proteins in mammals usually assemble into linear filaments.^50^, ^51^, ^52^, ^53^, ^54^, ^55^ They are members of the guanosine‐5′‐triphosphate (GTP)ase superfamily and have been implicated in numerous cellular processes, including membrane association, cell movement, cell polarity, and scaffold morphogenesis.^56^, ^57^ Septin 12 is expressed exclusively in the testis. Using gene targeting, it was demonstrated that *Sept* ^*+/−*^ chimeric mice were sterile with various sperm pathologies, including immotility, bent tails, acrosome breakage, and round heads. The *Sept12* chimeric mice also harbored significant DNA damage in their spermatozoa. The embryos that were generated by in vitro fertilization and ICSI failed to develop beyond the morula stage.^58^, ^59^ In 2012, two missense *SEPT12* (16p13.3) mutations, c.266C>T/p.Thr89Met and c.589G>A/p.Asp197Asn, were reported in infertile men.^60^ Both mutations are located inside the GTPase domain and might alter the protein structure, as suggested by in silico modeling. The p.Thr89Met mutation significantly reduced the hydrolytic activity of GTP and the p.Asp197Asn mutation interfered with GTP binding.^60^ Both forms of mutant SEPT12 proteins restricted filament formation by the wild‐type SEPT12 in a dose‐dependent manner. A patient who was carrying the p.Asp197Asn mutation presented with oligoasthenozoospermia, whereas a patient with the p.Thr89Met mutation had asthenoteratozoospermia. These findings suggest that loss‐of‐function mutations in *SEPT12* disrupt the structural integrity of sperm by perturbing septin filament formation.^60^

### 2.7 *TAF4B* and *ZMYND15* and recessive azoospermia

There were two unrelated consanguineous families with idiopathic azoospermia.^61^ In family 1, there were three azoospermic brothers and one oligozoospermic brother; in family 2, there were three azoospermic brothers.^61^ The candidate disease loci were found by linkage mapping by using data from single nucleotide polymorphism genome scans. Exome sequencing was applied to find the variants at these loci.^61^ In family 1, a non‐sense mutation, TATA box‐binding protein‐associated factor (*TAF*)*4B* (4p16.2) p.R611X, in exon 9 was deduced to truncate the protein product of the validated isoform by 252 residues (the native isoform has 862 amino acids).^61^ The truncated protein lacks the histone fold domain (residues 653‐702) that increases the DNA binding activity of TAFs,^62^ plus the interaction of the protein with TAF12, which is essential for DNA binding at the core promoters of several genes.^63^ *TAF4B*, also called “*TAFII105*” (RNA polymerase II, TATA box‐binding protein‐associated factor), has 15 exons and encodes an 862‐amino acid protein. It was found to have a predominant expression in the testis but very weak expression in the other organs and the authors found that the protein was enriched in mouse gonadal tissues.^64^ It was demonstrated that *Taf4b*‐null young mice initially were fertile but became infertile by 3 months, with impaired gonocyte proliferation and a reduced expression of spermatogonial stem cell markers, indicating that the gene protein is required for normal spermatogenic maintenance in adults.^65^ This gene has an additional feature in that the knockout female mice are also infertile because of folliculogenesis failure.^64^

In family 2, the mutation, zinc finger mynd‐containing protein 15 (*ZMYND15*) (17p22.1) p.K507Sfs*3, in exon 9 was deduced to shift the translational reading frame and lead to premature termination after the synthesis of two non‐native amino acids in all three validated protein isoforms: the mutant protein isoforms lacked 236 or 244 native residues.^61^ The truncated protein also lacked the Pro‐rich domain that is essential for the binding of some signal transduction and cytoskeletal proteins.^61^, ^66^ It was reported that *Zmynd15* in mice was expressed exclusively in the haploid germ cells.^67^ It also was found that *Zmynd15* expression was during late spermatogenesis and that the protein product was a transcriptional repressor that is essential for spermiogenesis.^67^

### 2.8 *NANOS1* and Sertoli‐cell‐only syndrome and oligoasthenoteratozoospermia

Nanos is a key translational regulator of specific mRNAs during *Drosophila* morphogenesis and germ cell development.^68^, ^69^, ^70^, ^71^, ^72^ It contains a conserved *C*‐terminal RNA‐binding zinc‐finger domain, while the *N*‐terminal region is responsible for binding protein partners.^73^ The resulting specific ribonucleoprotein complexes that contain nanos stimulate or repress translation. Whereas, the nanos protein in the fly is encoded by a single‐copy gene, three nanos homologs (Nanos1, Nanos2, and Nanos3) are present in mammals, including humans. All three are specifically expressed in the germ cells of adult men.^74^, ^75^ The disruption of the *Nanos2* and *Nanos3* genes in the mouse causes male infertility. This reveals their distinct, non‐overlapping roles, which emerged during the evolution of reproduction‐controlling mechanisms.^75^, ^76^ Nonetheless, the screening of *NANOS2* and *NANOS3* in a large group of men who were suffering from reproductive failure provided no evidence of infertility‐causing mutations of these two genes.^77^, ^78^ Therefore, although it has been shown that *Nanos1* disruption had no detrimental consequences for germ cell development in the mouse,^79^ the *NANOS1* (10q26.11) gene in this group of patients was analyzed. A group of 195 patients who manifested non‐obstructive azoospermia or oligozoospermia was tested for mutations in *NANOS1*, using single‐strand conformation polymorphism analysis and DNA sequencing.^80^ Three types of gene mutations were identified in five patients but they were absent in the 800 chromosomes of fertile men. The pedigree analysis indicated a dominant inheritance pattern with penetration limited to the male participants. Two mutations caused deletions of single amino acids, p.Pro77_Ser78delinsPro and p.Ala173del, each of them identified in two unrelated patients.^80^ It is known that a microRNA biogenesis factor, GEMIN3 protein, interacts with the *N*‐terminal region of NANOS1. Moreover, the NANOS1 and GEMIN3 proteins colocalize at the perinuclear region of male germ cells. At the developmental stage of round spermatids, both proteins were present within the chromatoid body.^81^ The Pro77_Ser78delinsPro mutation altered the interaction of NANOS1 with GEMIN3.^80^ The third identified mutation, p.([Arg246His; Arg276Tyr]), found in the *C*‐terminal RNA‐binding domain, was present in a single oligoasthenoteratozoospermic man. It was demonstrated that the p.Arg246His substitution caused a decrease in the positive charge of the domain, potentially altering RNA binding.^80^ These two different infertility phenotypes might reflect distinct functions of the *N*‐terminal, compared to the *C*‐terminal, region of NANOS1.

### 2.9 *GALNTL5* and asthenozoospermia

The glycosyltransferase‐like gene, human polypeptide *N*‐acetylgalactosaminyltransferase‐like protein 5 (*GALNTL5*)—also described as pp‐GalNac‐T19^82^ or GalNac‐T20^83^—belongs to the polypeptide *N*‐acetylgalactosaminyltransferase gene family because of its conserved glycosyltransferase domains, but it is uniquely truncated at the *C*‐terminal domain and is expressed exclusively in the human testis.^84^, ^85^, ^86^ In order to investigate the biological role of human *GALNTL5* (7q36.1) in the testis, the localization of the mouse orthologous protein, GALNTL5, was observed in spermatogenesis and *Galntl5*‐deficient mice were established.^87^ The heterozygous mutant male mice were infertile because of their immotile spermatozoa, a feature that corresponded to human azoospermia.^87^ It also was reported that the heterozygous mutation of *Galntl5* in mice impaired the amounts of glycolytic enzymes that are required for motility, protein loading into the acrosomes, and localization of the ubiquitin–proteasome system in the neck region in the spermatozoa, which consists of common cytoplasmic constituents that are shared among the haploid spermatids through intracellular bridges.^88^, ^89^ Screening for male patients with infertility that was caused by a mutation of the *GALNTL5* gene was conducted and one patient was identified among 200 whose asthenozoospermia was attributable to a heterozygous single nucleotide deletion of maternal inheritance in the exon of *GALNTL5*.^87^ This strongly suggests that the genetic mutation of human *GALNTL5* results in male infertility, with a reduction of sperm motility, and that GALNTL5 is a protein that is essential for mammalian sperm formation.

### 2.10 *PLK4* and Sertoli‐cell‐only syndrome

Polo‐like kinase 4 (PLK4) is the most structurally divergent member of the Polo protein family.^90^, ^91^, ^92^ Mice with a heterozygous mutation in *Plk4* frequently develop tumors in the liver and other organs.^93^ The PLK4 protein is a key regulator of the centriolar duplication that is required for the precise reproduction of centrosomes during the cell cycle.^94^ A heterozygous *Plk4* mutation (p.Ile242Asn) in mice caused patchy germ cell loss in the testes,^95^ similar to human Sertoli‐cell‐only syndrome (SCOS). The authors performed a direct PCR sequencing analysis of *PLK4* and this revealed a heterozygous 13 bp deletion in one patient: c.201_213delGAAACATCCTTCT. The other patients all showed normal sequences.^96^ Among 12 clones of PCR products that were expected to harbor the mutation, six (50%) showed the wild‐type allele and the other six carried the mutant allele. The heterozygous deletion (13 bp) results in a frame shift with a premature stop codon in the Ser/Thr kinase domain (*p.Lys68Serfs71*), excluding most of the Ser/Thr kinase domain and the whole polo‐box domain.^96^ The normal controls revealed no variant. The PLK4 protein is needed to phosphorylate substrates at the centrosome in order to promote centriolar duplication.^94^ Excessive PLK4 activity results in centriolar multiplication; however, catalytically inactive PLK4 (p.Asp154Ala) does not.^94^ The authors’ study demonstrated that the overexpression of the wild‐type PLK4 increased the number of centrioles in the transfected cells. By contrast, the overexpression of mutant PLK4 did not do so, compared with the wild‐type protein.^96^ The mutant PLK4 lost its Polo box and was not localized to the centrosome, consistent with the fact that the upstream sequence of the *C*‐terminal Polo box is required for this.^94^, ^97^ Morphological abnormalities of the nuclei were observed significantly more frequently in the cells that were transfected with mutant *PLK4*, compared with those that expressed the wild‐type gene and with the control cells.^96^ In addition, the nuclei in the cells that were transfected with the mutant *PLK4* were larger than those of the control cells and of those harboring the wild‐type *PLK4*.^96^ The study found that a mutation of *PLK4* at 4q28 caused SCOS in an infertile man. Centriolar duplication, occurring once every cell cycle, is controlled by *PLK4*. Haploinsufficiency of this gene can lead to a lack of centrioles, whereas overexpression increases them. In fact, the number of centrioles in the transfected cells was not changed by the overexpression of the mutant *PLK4* (13 bp deletion), compared with the controls. Dysregulation of *PLK4* should be harmful for the maintenance of chromosome stability by disturbing centrosomal duplication.^98^ It was demonstrated that the proportion of mitotic cells increased in the double mutant *Plk4* ^*−*/*−*^ mouse embryos, compared with the wild‐type ones at embryo day (E)7.5, suggesting that the completion of cell division was delayed or blocked.^99^ The morphological abnormalities that were observed in the nuclei of the cells that had been transfected with the mutant *PLK4* could imply mitotic errors. PLK4 acts as a tumor suppressor in hepatocellular carcinoma (HCC).^100^ PLK4 production is reduced during hepatic carcinogenesis by promoter hypermethylation^101^ and might contribute to HCC development in infertile men who carry a mutation in PLK4.^98^, ^102^

## 3 Culprit Genes That Have Been Identified in the X Chromosome

### 3.1 *TEX11* and azoospermia that is caused by meiotic arrest

*TEX11* contains a meiosis‐specific domain (SPO22) and numerous translocated promoter regions (TPRs) of unknown function. The TPRs are protein–protein interaction modules that are composed of helix–turn‐helix repeats that typically appear in tandem and pack with each other to form superhelical structures with various curvatures that can provide docking surfaces for other molecules. TEX11 is present in the DNA break–repair MRN complex (MRE11 [meiotic recombination 11]‐RAD50‐NBS1[Nijmegen breakage syndrome protein 1]).^103^ It regulates homologous chromosome synapsis and double‐strand DNA breakage repair. Thus, it is critical for synaptonemal complex and chiasma formation during chromosomal cross‐over.^103^, ^104^, ^105^, ^106^ The protein is highly conserved and functionally uniform across species.^103^ It was reported that male *Tex11*‐knockout mice showed infertility, with azoospermia that was caused by meiotic arrest.^107^ In 2015, a whole‐genome array comparative genomic hybridization screening study that involved 15 patients with azoospermia was conducted.^108^ A recurrent 99 kb *TEX11* intragenic deletion (Xq13.2) was identified in two unrelated patients. The deletion genomic region was rich in transposable elements and was prone to genomic rearrangements, as shown by a clustering of breakpoints of multiple duplications around TEX11 introns 9 and 12.^109^, ^110^, ^111^, ^112^ This loss, which was identical in both the patients with azoospermia, predicts a deletion of 79 amino acids within the SPO22 region. Mutation screening was performed by means of direct Sanger sequencing of the *TEX11* open reading frame in the blood and semen samples that had been obtained from 289 patients with azoospermia and from 384 controls.^108^ This showed five novel *TEX11* mutations: three splicing mutations and two missense mutations. These mutations, which occurred in seven of the men with azoospermia, were absent in the normal controls. Notably, five of those *TEX11* mutations were detected in 33 patients with azoospermia that was caused by meiotic arrest.^108^ An immunohistochemical analysis showed specific cytoplasmic TEX11 protein expression in the late spermatocytes and in the round and elongated spermatids in normal human testes. In contrast, the testes of the patients who suffered from azoospermia with *TEX11* mutations showed meiotic arrest and lacked TEX11 expression.^108^

## 4 Conclusion

Infertility is a serious social problem in advanced nations, with male‐factor infertility accounting for a significant proportion of the overall infertility among affected couples. Striking progress has been made in showing the mechanisms of spermatogenesis by using knockout mouse models. However, although many factors that are associated with male infertility are known in mice, the knockout mouse phenotypes do not necessarily have true human counterparts and the translation of the findings from the model organisms to humans has been slow. In vitro fertilization is often an efficient way to resolve infertility that is associated with female factors, but it is not very effective for severe male infertility. Microdissection testicular sperm extraction (MD‐TESE), coupled with ICSI, is very effective at finding and using the residual spermatozoa in the testes of men with azoospermia. However, MD‐TESE–ICSI cannot benefit patients when spermatogenesis is completely absent. Infertility that is caused by non‐obstructive azoospermia is very important in the field of assisted reproductive technology. Even with the aid of chromosomal analysis, ultrasonography of the testis, and detailed endocrinology, only MD‐TESE can confirm the presence of immature spermatozoa in the testes. The authors strongly hope that these studies will help clinics to avoid ineffective MD‐TESE procedures.

## Disclosures

*Conflict of interest*: The authors declare no conflict of interest. *Human and Animal Rights*: This article does not contain any study with human or animal participants that have been performed by any of the authors.

## References

[1] Di Spiezio Sardo A , Di Carlo C , Minozzi S , et al. Efficacy of hysteroscopy in improving reproductive outcomes of infertile couples: a systematic review and meta‐analysis. Hum Reprod Update. 2016;22:479‐496. 27008893 10.1093/humupd/dmw008

[2] Stahl PJ , Schlegel PN . Genetic evaluation of the azoospermic or severely oligozoospermic male. Curr Opin Obstet Gynecol. 2012;24:221‐228. 22729088 10.1097/GCO.0b013e3283558560

[3] Miyamoto T , Minase G , Okabe K , Ueda H , Sengoku K . Male infertility and its genetic causes. J Obstet Gynaecol Res. 2015;41:1501‐1505. 26178295 10.1111/jog.12765

[4] Reijo R , Lee TY , Salo P , et al. Diverse spermatogenic defects in humans caused by Y chromosome deletions encompassing a novel RNA‐binding protein gene. Nat Genet. 1995;10:383‐393. 7670487 10.1038/ng0895-383

[5] Matzuk MM , Lamb DJ . The biology of infertility: research advances and clinical challenges. Nat Med. 2008;14:1197‐1213. 18989307 10.1038/nm.f.1895 PMC3786590

[6] Elliott DJ , Millar MR , Oghene K , et al. Expression of RBM in the nuclei of human germ cells is dependent on a critical region of the Y chromosome long arm. Proc Natl Acad Sci USA. 1997;94:3848‐3853. 9108067 10.1073/pnas.94.8.3848 PMC20530

[7] Sun C , Skaletsky H , Birren B , et al. An azoospermic man with a de novo point mutation in the Y‐chromosomal gene USP9Y. Nat Genet. 1999;23:429‐432. 10581029 10.1038/70539

[8] Krausz C , Hoefsloot L , Simoni M , Tüttelmann F . European Academy of Andrology, European Molecular Genetics Quality Network . EAA/EMQN best practice guidelines for molecular diagnosis of Y‐chromosomal microdeletions: state‐of‐the‐art 2013. Andrology. 2014;2:5‐19. 24357628 10.1111/j.2047-2927.2013.00173.x PMC4065365

[9] Suganthi R , Vijesh VV , Vandana N , Fathima Ali Benazir J . Y choromosomal microdeletion screening in the workup of male infertility and its current status in India. Int J Fertil Steril. 2014;7:253‐266. 24520494 PMC3901175

[10] Miyamoto T , Hasuike S , Yogev L , et al. Azoospermia in patients heterozygous for a mutation in SYCP3. Lancet. 2003;362:1714‐1719. 14643120 10.1016/S0140-6736(03)14845-3

[11] Yuan L , Liu JG , Zhao J , Brundell E , Daneholt B , Hoog C . The murine SCP3 gene is required for synaptonemal complex assembly, chromosome synapsis, and male fertility. Mol Cell. 2000;5:73‐83. 10678170 10.1016/s1097-2765(00)80404-9

[12] Dobson MJ , Pearlman RE , Karaiskakis A , Spyropoulos B , Moens PB . Synaptonemal complex proteins: occurrence, epitope mapping and chromosome disjunction. J Cell Sci. 1994;107:2749‐2760. 7876343 10.1242/jcs.107.10.2749

[13] Lammers JH , Offenberg HH , van Aalderen M , Vink AC , Dietrich AJ , Heyting C . The gene encoding a major component of the lateral elements of synaptonemal complexes of the rat is related to X‐linked lymphocyte‐regulated genes. Mol Cell Biol. 1994;14:1137‐1146. 8289794 10.1128/mcb.14.2.1137-1146.1994 PMC358469

[14] Yuan L , Pelttari J , Brundell E , et al. The synaptonemal complex protein SCP3 can form multistranded, cross‐striated fibers in vivo. J Cell Biol. 1998;142:331‐339. 9679134 10.1083/jcb.142.2.331 PMC2133048

[15] Yuan L , Liu JG , Hoja MR , Wilbertz J , Nordqvist K , Höög C . Female germ cell aneuploidy and embryo death in mice lacking the meiosis‐specific protein SCP3. Science. 2002;296:1115‐1118. 12004129 10.1126/science.1070594

[16] Meuwissen RL , Offenberg HH , Dietrich AJ , Riesewijk A , van Iersel M , Heyting C . A coiled‐coil related protein specific for synapsed regions of meiotic prophase chromosomes. EMBO J. 1992;11:5091‐5100. 1464329 10.1002/j.1460-2075.1992.tb05616.x PMC556987

[17] Bolor H , Mori T , Nishiyama S , et al. Mutations of the SYCP3 gene in women with recurrent pregnancy loss. Am J Hum Genet. 2009;84:14‐20. 19110213 10.1016/j.ajhg.2008.12.002 PMC2668043

[18] Yatsenko AN , Roy A , Chen R , et al. Non‐invasive genetic diagnosis of male infertility using spermatozoal RNA: KLHL10 mutations in oligozoospermic patients impair homodimerization. Hum Mol Genet. 2006;15:3411‐3419. 17047026 10.1093/hmg/ddl417

[19] Wang S , Zheng H , Esaki Y , Kelly F , Yan W . Cullin3 is a KLHL10‐interacting protein preferentially expressed during late spermiogenesis. Biol Reprod. 2006;74:102‐108. 16162871 10.1095/biolreprod.105.045484

[20] Yan W , Ma L , Burns KH , Matzuk MM . Haploinsufficiency of kelch‐like protein homolog 10 causes infertility in male mice. Proc Natl Acad Sci USA. 2004;101:7793‐7798. 15136734 10.1073/pnas.0308025101 PMC419685

[21] Dam AH , Feenstra I , Westphal JR , Ramos L , van Golde RJ , Kremer JA . Globozoospermia revisited. Hum Reprod Update. 2007;13:63‐75. 17008355 10.1093/humupd/dml047

[22] Dale B , Iaccarino M , Fortunato A , Gragnaniello G , Kyozuka K , Tosti E . A morphological and functional study of fusibility in round‐headed spermatozoa in the human. Fertil Steril. 1994;61:336‐340. 8299793 10.1016/s0015-0282(16)56528-5

[23] Flörke‐Gerloff S , Töpfer‐Petersen E , Müller‐Esterl W , et al. Biochemical and genetic investigation of round‐headed spermatozoa in infertile men including two brothers and their father. Andrologia. 1984;16:187‐202. 6380341 10.1111/j.1439-0272.1984.tb00262.x

[24] Nistal M , Paniagua R , Herruzo A . Multi‐tailed spermatozoa in a case with asthenospermia and teratospermia. Virchows Arch B Cell Pathol. 1977;26:111‐118. 415424 10.1007/BF02889540

[25] Escalier D . Human spermatozoa with large heads and multiple flagella: a quantitative ultrastructural study of 6 cases. Biol Cell. 1983;48:65‐74. 6673791 10.1111/j.1768-322x.1984.tb00203.x

[26] Escalier D . Genetic approach to male meiotic division deficiency: the human macronuclear spermatozoa. Mol Hum Reprod. 2002;8:1‐7. 11756563 10.1093/molehr/8.1.1

[27] Dieterich K , Soto Rifo R , Faure AK , et al. Homozygous mutation of AURKC yields large‐headed polyploid spermatozoa and causes male infertility. Nat Genet. 2007;39:661‐665. 17435757 10.1038/ng2027

[28] Bernard M , Sanseau P , Henry C , Couturier A , Prigent C . Cloning of STK13, a third human protein kinase related to Drosophila aurora and budding yeast Ipl1 that maps on chromosome 19q13.3‐ter. Genomics. 1998;53:406‐409. 9799611 10.1006/geno.1998.5522

[29] Kimura M , Matsuda Y , Yoshioka T , Okano Y . Cell cycle‐dependent expression and centrosome localization of a third human aurora/Ipl1‐related protein kinase, AIK3. J Biol Chem. 1999;274:7334‐7340. 10066797 10.1074/jbc.274.11.7334

[30] Tang CJ , Chuang CK , Hu HM , Tang TK . The zinc finger domain of Tzfp binds to the tbs motif located at the upstream flanking region of the Aie1 (aurora‐C) kinase gene. J Biol Chem. 2001;276:19631‐19639. 11279021 10.1074/jbc.M100170200

[31] Tang CJ , Lin CY , Tang TK . Dynamic localization and functional implications of Aurora‐C kinase during male mouse meiosis. Dev Biol. 2006;290:398‐410. 16386730 10.1016/j.ydbio.2005.11.036

[32] Ben Khelifa M , Zouari R , Harbuz R , et al. A new AURKC mutation causing macrozoospermia: implications for human spermatogenesis and clinical diagnosis. Mol Hum Reprod. 2011;17:762‐768. 21733974 10.1093/molehr/gar050 PMC3639514

[33] Ben Khelifa M , Coutton C , Blum MG , et al. Identification of a new recurrent aurora kinase C mutation in both European and African men with macrozoospermia. Hum Reprod. 2012;27:3337‐3346. 22888167 10.1093/humrep/des296

[34] Kilani Z , Ismail R , Ghunaim S , et al. Evaluation and treatment of familial globozoospermia in five brothers. Fertil Steril. 2004;82:1436‐1439. 15533374 10.1016/j.fertnstert.2004.03.064

[35] Xu X , Toselli PA , Russell LD , Seldin DC . Globozoospermia in mice lacking the casein kinase II alpha’ catalytic subunit. Nat Genet. 1999;23:118‐121. 10471512 10.1038/12729

[36] Kang‐Decker N , Mantchev GT , Juneja SC , McNiven MA , van Deursen JM . Lack of acrosome formation in Hrb‐deficient mice. Science. 2001;294:1531‐1533. 11711676 10.1126/science.1063665

[37] Yao R , Ito C , Natsume Y , et al. Lack of acrosome formation in mice lacking a Golgi protein, GOPC. Proc Natl Acad Sci USA. 2002;99:11211‐11216. 12149515 10.1073/pnas.162027899 PMC123235

[38] Dam AH , Koscinski I , Kremer JA , et al. Homozygous mutation in SPATA16 is associated with male infertility in human globozoospermia. Am J Hum Genet. 2007;81:813‐820. 17847006 10.1086/521314 PMC2227931

[39] Karaca N , Yilmaz R , Kanten GE , Kervancioglu E , Solakoglu S , Kervancioglu ME . First successful pregnancy in a globozoospermic patient having homozygous mutation in SPATA16. Fertil Steril. 2014;102:103‐107. 24825417 10.1016/j.fertnstert.2014.04.002

[40] Hu Z , Xia Y , Guo X , et al. A genome‐wide association study in Chinese men identifies three risk loci for non‐obstructive azoospermia. Nat Genet. 2011;44:183‐186. 22197933 10.1038/ng.1040

[41] Goodson ML , Park‐Sarge OK , Sarge KD . Tissue‐dependent expression of heat shock factor 2 isoforms with distinct transcriptional activities. Mol Cell Biol. 1995;15:5288‐5293. 7565677 10.1128/mcb.15.10.5288 PMC230776

[42] Sarge KD , Cullen KE . Regulation of hsp expression during rodent spermatogenesis. Cell Mol Life Sci. 1997;53:191‐197. 9118007 10.1007/PL00000591 PMC11147273

[43] Abane R , Mezger V . Roles of heat shock factors in gametogenesis and development. FEBS J. 2010;277:4150‐4172. 20945531 10.1111/j.1742-4658.2010.07830.x

[44] Dix DJ , Allen JW , Collins BW , et al. Targeted gene disruption of Hsp70‐2 results in failed meiosis, germ cell apoptosis, and male infertility. Proc Natl Acad Sci USA. 1996;93:3264‐3268. 8622925 10.1073/pnas.93.8.3264 PMC39594

[45] Dix DJ , Allen JW , Collins BW , et al. HSP70‐2 is required for desynapsis of synaptonemal complexes during meiotic prophase in juvenile and adult mouse spermatocytes. Development. 1997;124:4595‐4603. 9409676 10.1242/dev.124.22.4595

[46] Kallio M , Chang Y , Manuel M , et al. Brain abnormalities, defective meiotic chromosome synapsis and female subfertility in HSF2 null mice. EMBO J. 2002;21:2591‐2601. 12032072 10.1093/emboj/21.11.2591 PMC125382

[47] Wang G , Zhang J , Moskophidis D , Mivechi NF . Targeted disruption of the heat shock transcription factor (hsf)‐2 gene results in increased embryonic lethality, neuronal defects, and reduced spermatogenesis. Genesis. 2003;36:48‐61. 12748967 10.1002/gene.10200

[48] Wang G , Ying Z , Jin X , et al. Essential requirement for both hsf1 and hsf2 transcriptional activity in spermatogenesis and male fertility. Genesis. 2004;38:66‐80. 14994269 10.1002/gene.20005

[49] Mou L , Wang Y , Li H , et al. A dominant‐negative mutation of HSF2 associated with idiopathic azoospermia. Hum Genet. 2013;132:159‐165. 23064888 10.1007/s00439-012-1234-7

[50] Fujishima K , Kiyonari H , Kurisu J , Hirano T , Kengaku M . Targeted disruption of Sept3, a heteromeric assembly partner of Sept5 and Sept7 in axons, has no effect on developing CNS neurons. J Neurochem. 2007;102:77‐92. 17564677 10.1111/j.1471-4159.2007.04478.x

[51] Joberty G , Perlungher RR , Sheffield PJ , et al. Borg proteins control septin organization and are negatively regulated by Cdc42. Nat Cell Biol. 2001;3:861‐866. 11584266 10.1038/ncb1001-861

[52] Lukoyanova N , Baldwin SA , Trinick J . 3D reconstruction of mammalian septin filaments *J* . Mol Biol. 2008;376:1‐7. 10.1016/j.jmb.2007.11.029 18083193

[53] Nagata K , Asano T , Nozawa Y , Inagaki M . Biochemical and cell biological analyses of a mammalian septin complex, Sept7/9b/11. J Biol Chem. 2004;279:55895‐55904. 15485874 10.1074/jbc.M406153200

[54] Sirajuddin M , Farkasovsky M , Hauer F , et al. Structural insight into filament formation by mammalian septins. Nature. 2007;449:311‐315. 17637674 10.1038/nature06052

[55] Xie Y , Vessey JP , Konecna A , Dahm R , Macchi P , Kiebler MA . The GTP‐binding protein Septin 7 is critical for dendrite branching and dendritic‐spine morphology. Curr Biol. 2007;17:1746‐1751. 17935997 10.1016/j.cub.2007.08.042

[56] Barral Y , Kinoshita M . Structural insights shed light onto septin assemblies and function. Curr Opin Cell Biol. 2008;20:12‐18. 18242072 10.1016/j.ceb.2007.12.001

[57] Hall PA , Russell SE . The pathobiology of the septin gene family. J Pathol. 2004;204:489‐505. 15495264 10.1002/path.1654

[58] Lin YH , Lin YM , Wang YY , et al. The expression level of septin12 is critical for spermiogenesis. Am J Pathol. 2009;174:1857‐1868. 19359518 10.2353/ajpath.2009.080955 PMC2671274

[59] Lin YH , Chou CK , Hung YC , et al. SEPT12 deficiency causes sperm nucleus damage and developmental arrest of preimplantation embryos. Fertil Steril. 2011;95:363‐365. 20801438 10.1016/j.fertnstert.2010.07.1064

[60] Kuo YC , Lin YH , Chen HI , et al. SEPT12 mutations cause male infertility with defective sperm annulus. Hum Mutat. 2012;33:710‐719. 22275165 10.1002/humu.22028

[61] Ayhan Ö , Balkan M , Guven A , et al. Truncating mutations in TAF4B and ZMYND15 causing recessive azoospermia. J Med Genet. 2014;51:239‐244. 24431330 10.1136/jmedgenet-2013-102102

[62] Shao H , Revach M , Moshonov S , et al. Core promoter binding by histone‐like TAF complexes. Mol Cell Biol. 2005;25:206‐219. 15601843 10.1128/MCB.25.1.206-219.2005 PMC538770

[63] Gazit K , Moshonov S , Elfakess R , et al. TAF4/4b × TAF12 displays a unique mode of DNA binding and is required for core promoter function of a subset of genes. J Biol Chem. 2009;284:26286‐26296. 19635797 10.1074/jbc.M109.011486 PMC2785316

[64] Freiman RN , Albright SR , Zheng S , Sha WC , Hammer RE , Tjian R . Requirement of tissue‐selective TBP‐associated factor TAFII105 in ovarian development. Science. 2001;293:2084‐2087. 11557891 10.1126/science.1061935

[65] Falender AE , Freiman RN , Geles KG , et al. Maintenance of spermatogenesis requires TAF4b, a gonad‐specific subunit of TFIID. Genes Dev. 2005;19:794‐803. 15774719 10.1101/gad.1290105 PMC1074317

[66] Kay BK , Williamson MP , Sudol M . The importance of being proline: the interaction of proline‐rich motifs in signaling proteins with their cognate domains. FASEB J. 2000;14:231‐241. 10657980

[67] Yan W , Si Y , Slaymaker S , et al. Zmynd15 encodes a histone deacetylase‐dependent transcriptional repressor essential for spermiogenesis and male fertility. J Biol Chem. 2010;285:31418‐31426. 20675388 10.1074/jbc.M110.116418 PMC2951216

[68] Kobayashi S , Yamada M , Asaoka M , Kitamura T . Essential role of the posterior morphogen nanos for germline development in Drosophila. Nature. 1996;380:708‐711. 8614464 10.1038/380708a0

[69] Asaoka M , Sano H , Obara Y , Kobayashi S . Maternal Nanos regulates zygotic gene expression in germline progenitors of Drosophila melanogaster. Mech Dev. 1998;78:153‐158. 9858716 10.1016/s0925-4773(98)00164-6

[70] Forbes A , Lehmann R . Nanos and Pumilio have critical roles in the development and function of Drosophila germline stem cells. Development. 1998;125:679‐690. 9435288 10.1242/dev.125.4.679

[71] Asaoka‐Taguchi M , Yamada M , Nakamura A , Hanyu K , Kobayashi S . Maternal Pumilio acts together with Nanos in germline development in Drosophila embryos. Nat Cell Biol. 1999;1:431‐437. 10559987 10.1038/15666

[72] Bhat KM . The posterior determinant gene nanos is required for the maintenance of the adult germline stem cells during Drosophila oogenesis. Genetics. 1999;151:1479‐1492. 10101171 10.1093/genetics/151.4.1479 PMC1460561

[73] Verrotti AC , Wharton RP . Nanos interacts with cup in the female germline of Drosophila. Development. 2000;127:5225‐5232. 11060247 10.1242/dev.127.23.5225

[74] Jaruzelska J , Kotecki M , Kusz K , Spik A , Firpo M , Reijo Pera RA . Conservation of a Pumilio‐Nanos complex from Drosophila germ plasm to human germ cells. Dev Genes Evol. 2003;213:120‐126. 12690449 10.1007/s00427-003-0303-2

[75] Tsuda M , Sasaoka Y , Kiso M , et al. Conserved role of nanos proteins in germ cell development. Science. 2003;29:1239‐1241. 10.1126/science.1085222 12947200

[76] Suzuki A , Tsuda M , Saga Y . Functional redundancy among Nanos proteins and a distinct role of Nanos2 during male germ cell development. Development. 2007;134:77‐83. 17138666 10.1242/dev.02697

[77] Kusz KM , Tomczyk L , Sajek M , et al. The highly conserved NANOS2 protein: testis‐specific expression and significance for the human male reproduction. Mol Hum Reprod. 2009;15:165‐171. 19168545 10.1093/molehr/gap003

[78] Kusz K , Tomczyk L , Spik A , et al. NANOS3 gene mutations in men with isolated sterility phenotype. Mol Reprod Dev. 2009;76:804. 19565640 10.1002/mrd.21070

[79] Haraguchi S , Tsuda M , Kitajima S , et al. nanos1: a mouse nanos gene expressed in the central nervous system is dispensable for normal development. Mech Dev. 2003;120:721‐731. 12834871 10.1016/s0925-4773(03)00043-1

[80] Kusz‐Zamelczyk K , Sajek M , Spik A , et al. Mutations of NANOS1, a human homologue of the Drosophila morphogen, are associated with a lack of germ cells in testes or severe oligo‐astheno‐teratozoospermia. J Med Genet. 2013;50:187‐193. 23315541 10.1136/jmedgenet-2012-101230

[81] Ginter‐Matuszewska B , Kusz K , Spik A , et al. NANOS1 and PUMILIO2 bind microRNA biogenesis factor GEMIN3, within chromatoid body in human germ cells. Histochem Cell Biol. 2011;136:279‐287. 21800163 10.1007/s00418-011-0842-y

[82] Peng C , Togayachi A , Kwon YD , et al. Identification of a novel human UDP‐GalNAc transferase with unique catalytic activity and expression profile. Biochem Biophys Res Commun. 2010;402:680‐686. 20977886 10.1016/j.bbrc.2010.10.084

[83] Raman J , Guan Y , Perrine CL , Gerken TA , Tabak LA . UDP‐N‐acetyl‐α‐D‐galactosamine: polypeptide N‐acetylgalactosaminyltransferases: completion of the family tree. Glycobiology. 2012;22:768‐777. 22186971 10.1093/glycob/cwr183 PMC3336867

[84] Bennett EP , Hassan H , Clausen H . cDNA cloning and expression of a novel human UDP‐N‐acetyl‐alpha‐D‐galactosamine. Polypeptide N‐acetylgalactosaminyltransferase, GalNAc‐t3. J Biol Chem. 1996;271:17006‐17012. 8663203 10.1074/jbc.271.29.17006

[85] White T , Bennett EP , Takio K , Sørensen T , Bonding N , Clausen H . Purification and cDNA cloning of a human UDP‐N‐acetyl‐alpha‐D‐galactosamine: polypeptide N‐acetylgalactosaminyltransferase. J Biol Chem. 1995;270:24156‐24165. 7592619 10.1074/jbc.270.41.24156

[86] Guo JM , Zhang Y , Cheng L , et al. Molecular cloning and characterization of a novel member of the UDP‐GalNAc: polypeptide N‐acetylgalactosaminyltransferase family, pp‐GalNAc‐T12. FEBS Lett. 2002;524:211‐218. 12135769 10.1016/s0014-5793(02)03007-7

[87] Takasaki N , Tachibana K , Ogasawara S , et al. A heterozygous mutation of GALNTL5 affects male infertility with impairment of sperm motility. Proc Natl Acad Sci USA. 2014;111:1120‐1125. 24398516 10.1073/pnas.1310777111 PMC3903224

[88] Ventelä S , Toppari J , Parvinen M . Intercellular organelle traffic through cytoplasmic bridges in early spermatids of the rat: mechanisms of haploid gene product sharing. Mol Biol Cell. 2003;14:2768‐2780. 12857863 10.1091/mbc.E02-10-0647 PMC165675

[89] Braun RE , Behringer RR , Peschon JJ , Brinster RL , Palmiter RD . Genetically haploid spermatids are phenotypically diploid. Nature. 1989;337:373‐376. 2911388 10.1038/337373a0

[90] Barr FA , Silljé HH , Nigg EA . Polo‐like kinases and the orchestration of cell division. Nat Rev Mol Cell Biol. 2004;5:429‐440. 15173822 10.1038/nrm1401

[91] Glover DM . Polo kinase and progression through M phase in Drosophila: a perspective from the spindle poles. Oncogene. 2005;24:230‐237. 15640838 10.1038/sj.onc.1208279

[92] Swallow CJ , Ko MA , Siddiqui NU , Hudson JW , Dennis JW . Sak/Plk4 and mitotic fidelity. Oncogene. 2005;24:306‐312. 15640847 10.1038/sj.onc.1208275

[93] Ko MA , Rosario CO , Hudson JW , et al. Plk4 haploinsufficiency causes mitotic infidelity and carcinogenesis. Nat Genet. 2005;37:883‐888. 16025114 10.1038/ng1605

[94] Habedanck R , Stierhof YD , Wilkinson CJ , Nigg EA . The Polo kinase Plk4 functions in centriole duplication. Nat Cell Biol. 2005;7:1140‐1146. 16244668 10.1038/ncb1320

[95] Harris RM , Weiss J , Jameson JL . Male hypogonadism and germ cell loss caused by a mutation in Polo‐like kinase 4. Endocrinology. 2011;152:3975‐3985. 21791561 10.1210/en.2011-1106 PMC3176650

[96] Miyamoto T , Bando Y , Koh E , et al. A PLK4 mutation causing azoospermia in a man with Sertoli cell‐only syndrome. Andrology. 2016;4:75‐81. 26452337 10.1111/andr.12113

[97] Leung GC , Hudson JW , Kozarova A , Davidson A , Dennis JW , Sicheri F . The Sak polo‐box comprises a structural domain sufficient for mitotic subcellular localization. Nat Struct Biol. 2002;9:719‐724. 12352953 10.1038/nsb848

[98] Rosario CO , Ko MA , Haffani YZ , et al. Plk4 is required for cytokinesis and maintenance of chromosome stability. Proc Natl Acad Sci USA. 2010;107:6888‐6893. 20348415 10.1073/pnas.0910941107 PMC2872425

[99] Hudson JW , Kozarova A , Cheung P , et al. Late mitotic failure in mice lacking Sak, polo‐like kinase. Curr Biol. 2001;11:441‐446. 11301255 10.1016/s0960-9822(01)00117-8

[100] Liu L , Zhang CZ , Cai M , Fu J , Chen GG , Yun J . Downregulation of polo‐like kinase 4 in hepatocellular carcinoma associates with poor prognosis. PLoS ONE. 2012;7:e41293. 22829937 10.1371/journal.pone.0041293 PMC3400587

[101] Ward A , Morettin A , Shum D , Hudson JW . Aberrant methylation of Polo‐like kinase CpG islands in Plk4 heterozygous mice. BMC Cancer. 2011;11:71. 21324136 10.1186/1471-2407-11-71 PMC3047422

[102] Pellegrino R , Calvisi DF , Ladu S , et al. Oncogenic and tumor suppressive roles of polo‐like kinases in human hepatocellular carcinoma. Hepatology. 2010;51:857‐868. 20112253 10.1002/hep.23467

[103] Adelman CA , Petrini JH . ZIP4H (TEX11) deficiency in the mouse impairs meiotic double strand break repair and the regulation of crossing over. PLoS Genet. 2008;4:e1000042. 18369460 10.1371/journal.pgen.1000042 PMC2267488

[104] Yang F , Gell K , van der Heijden GW , et al. Meiotic failure in male mice lacking an X‐linked factor. Genes Dev. 2008;22:682‐691. 18316482 10.1101/gad.1613608 PMC2259036

[105] Tsubouchi T , Zhao H , Roeder GS . The meiosis‐specific zip4 protein regulates crossover distribution by promoting synaptonemal complex formation together with zip2. Dev Cell. 2006;10:809‐819. 16740482 10.1016/j.devcel.2006.04.003

[106] Primig M , Williams RM , Winzeler EA , et al. The core meiotic transcriptome in budding yeasts. Nat Genet. 2000;26:415‐423. 11101837 10.1038/82539

[107] Perry J , Kleckner N , Börner GV . Bioinformatic analyses implicate the collaborating meiotic crossover/chiasma proteins Zip2, Zip3, and Spo22/Zip4 in ubiquitin labeling. Proc Natl Acad Sci USA. 2005;102:17594‐17599. 16314568 10.1073/pnas.0508581102 PMC1308910

[108] Yatsenko AN , Georgiadis AP , Röpke A , et al. X‐linked TEX11 mutations, meiotic arrest, and azoospermia in infertile men. N Engl J Med. 2015;372:2097‐2107. 25970010 10.1056/NEJMoa1406192 PMC4470617

[109] Mühle C , Zenker M , Chuzhanova N , Schneider H . Recurrent inversion with concomitant deletion and insertion events in the coagulation factor VIII gene suggests a new mechanism for X‐chromosomal rearrangements causing hemophilia A. Hum Mutat. 2007;28:1045. 10.1002/humu.9506 17823971

[110] Lakich D , Kazazian HH Jr , Antonarakis SE , Gitschier J . Inversions disrupting the factor VIII gene are a common cause of severe haemophilia A. Nat Genet. 1993;5:236‐241. 8275087 10.1038/ng1193-236

[111] Huang CR , Schneider AM , Lu Y , et al. Mobile interspersed repeats are major structural variants in the human genome. Cell. 2010;141:1171‐1182. 20602999 10.1016/j.cell.2010.05.026 PMC2943426

[112] Xing J , Zhang Y , Han K , et al. Mobile elements create structural variation: analysis of a complete human genome. Genome Res. 2009;19:1516‐1526. 19439515 10.1101/gr.091827.109 PMC2752133

